# Assessment of Patient Reported Outcomes (PROs) in Outpatients Taking Oral Anticancer Drugs Included in the Real-Life Oncoral Program

**DOI:** 10.3390/cancers14030660

**Published:** 2022-01-28

**Authors:** Bastien Collomb, Amélie Dubromel, Anne Gaëlle Caffin, Chloé Herledan, Virginie Larbre, Amandine Baudouin, Ariane Cerutti, Laurence Couturier, Magali Maire, Lionel Karlin, Delphine Maucort-Boulch, Laure Huot, Stéphane Dalle, Emmanuel Bachy, Hervé Ghesquieres, Gilles Salles, Sébastien Couraud, Benoit You, Gilles Freyer, Véronique Trillet-Lenoir, Florence Ranchon, Catherine Rioufol

**Affiliations:** 1 Unité de Pharmacie Clinique Oncologique, Groupement Hospitalier Sud, Hospices Civils de Lyon, 69495 Lyon, France; Bastien.COLLOMB@ch-le-vinatier.fr (B.C.); amelie.dubromel@chu-lyon.fr (A.D.); anne-gaelle.caffin@chu-lyon.fr (A.G.C.); chloe.herledan@chu-lyon.fr (C.H.); virginie.larbre@chu-lyon.fr (V.L.); amandine.baudouin@chu-lyon.fr (A.B.); ariane.cerutti@chu-lyon.fr (A.C.); laurence.couturier@chu-lyon.fr (L.C.); magali.maire@chu-lyon.fr (M.M.); florence.ranchon@chu-lyon.fr (F.R.); 2 CICLY Centre pour l’Innovation en Cancérologie de Lyon, Université Lyon 1-EA 3738, 69921 Lyon, France; sebastien.couraud@chu-lyon.fr (S.C.); benoit.you@chu-lyon.fr (B.Y.); gilles.freyer@chu-lyon.fr (G.F.); veronique.trillet-lenoir@chu-lyon.fr (V.T.-L.); 3 Department of Hematology, Groupement Hospitalier Sud, Hospices Civils de Lyon, 69495 Lyon, France; Lionel.karlin@chu-lyon.fr (L.K.); emmanuel.bachy@chu-lyon.fr (E.B.); herve.ghesquieres@chu-lyon.fr (H.G.); gilles.salles@chu-lyon.fr (G.S.); 4 Department of Statistics and Bioinformatics, Hospices Civils de Lyon, 69002 Lyon, France; delphine.maucort-boulch@chu-lyon.fr; 5 CNRS UMR 5558, Laboratoire de Biométrie et Biologie Evolutive, Equipe Biostatistique-Santé, 69622 Lyon, France; 6 Cellule Innovation, Département de la Recherche Clinique et de L’innovation, Hospices Civils de Lyon, 69002 Lyon, France; laure.huot@chu-lyon.fr; 7 Dermatology Department, Groupement Hospitalier Lyon Sud, Hospices Civils de Lyon, 69495 Lyon, France; stephane.dalle@chu-lyon.fr; 8 Department of Pneumology, Groupement Hospitalier Sud, Hospices Civils de Lyon, 69495 Lyon, France; 9 Department of Oncology, Groupement Hospitalier Sud, Hospices Civils de Lyon, 69495 Lyon, France

**Keywords:** patient reported outcomes (PROs), patient reported outcomes measures (PROMs), ambulatory care, oral anticancer agent (OAA)

## Abstract

**Simple Summary:**

Patients receiving oral anticancer agents (OAAs) have a substantial symptom burden. Given the trend toward patient-centered care, the use of patient-reported outcomes (PROs) seems appropriate to secure medication management, and to improve clinical decision-making. The aim of this study was to assess symptoms potentially related to adverse events experienced by cancer outpatients treated by OAAs using PROs. In total, 407 questionnaires were completed by 63 patients, in which 2333 symptoms were reported. Almost three-quarters (74.6%) reported at least one high-level symptom. The symptoms most commonly experienced were fatigue, various psychological disorders and general pain. This initiative is the first step in the implementation of symptom assessment by PROs in patients treated by OAAs. The results highlight the need for close coordination between community and hospital health professionals, and the integration of patient self-reporting systems in oncologic clinical practice.

**Abstract:**

Background In previous studies, patient-reported outcomes (PROs) have been shown to improve survival in cancer patients. The aim of the present study was to assess symptoms potentially related to adverse events experienced by cancer outpatients treated by oral anticancer agents (OAAs) using PROs. Methods Between September 2018 and May 2019, outpatients starting OAAs were included in a 12-week follow-up to assess 15 symptoms listed in the National Cancer Institute PRO Common Terminology Criteria for Adverse Events, using a 5-point scale of severity or frequency. Patients were requested to alert a referral nurse or pharmacist when they self-assessed high-level (level 3 or 4) symptoms. Results 407 questionnaires were completed by 63 patients in which 2333 symptoms were reported. Almost three-quarters (74.6%) reported at least one high-level symptom. The symptoms that were most commonly experienced were fatigue (>9 in 10 patients; 13.2% of symptoms declared), various psychological disorders (>9 in 10 patients; 28.6% of symptoms declared) and general pain (>8 in 10 patients; 9.4% of symptoms declared). Conclusion PROs are appropriate to detect potential adverse events in cancer outpatients treated by OAAs. This study is the first step for integrating the patient’s perspective in a digital e-health device in routine oncology care.

## 1. Introduction

The approach to cancer treatment has been changing in the last two decades due to the increasing number of Oral Anticancer Agents (OAAs); in the United States, more than a quarter of the antineoplastic agents under development in the early 2000s were planned as oral drugs, and in 2019 more than half of all FDA (Food and Drugs Administration)-approved cancer treatments were oral [1,2]. As well as a great improvement in disease outcome, OAAs are a major shift for patients, who can now manage their cancer in an outpatient setting, offering better quality of life by making the treatments coexist with everything else in the patient’s life; occupation or school, family life, social life [3]. The growing number of OAAs significantly impacts all aspects of oncology, including healthcare spending, patient-physician relationship, treatment adherence, and side-effects monitoring [1,4].

Symptoms and treatment-related toxicity in patients receiving OAAs, traditionally reported by physicians, are increasingly also assessed by the patients themselves [5]. Firstly, medical staff may underestimate or fail to recognize symptom severity as compared to the patient’s self-perception [6,7,8,9]. Secondly, understanding patient perception is now seen to be of considerable importance for improving cancer management [10]. One approach to facilitate patient-centered care is the use of patient-reported outcomes (PROs) in clinical practice. PROs are defined by the FDA as a “measurement based on a report that comes directly from the patient about the status of a patient’s health condition, without amendment or interpretation of the patient’s response by a clinician or anyone else” [11]. PRO assessment was initially developed in clinical trials, as a standard data source to capture the patient’s subjective experience, usually as a secondary endpoint [12]. It is now currently used as the primary endpoint [13,14].

As part of the shift to patient-centered care, including PROs in clinical practice has become a subject of growing interest for stakeholders. The potential benefits of PROs have been previously described: better patient-provider communication and clinical decision-making; timely reporting and management of symptoms; less patient anxiety; fewer preventable emergency room and office visits or calls; better patient adherence to advice; greater satisfaction with care; less litigation; more effective self-management; and more efficient use of resources [15,16]. PRO collection includes standardized validated generic or disease-specific tools, known as patient-reported outcomes measures (PROMs). Several tools have been developed, the most widely used being the first version of the U.S. National Cancer Institute‘s (NCI) patient-reported outcomes version of the Common Terminology Criteria for Adverse Events (PRO-CTCAE v1.0) [17]. The general approach includes a brief survey, administered in most cases via the internet, an app or an automated telephone system, with alerts to clinicians for worrying or worsening issues. Patients are generally asked to self-report on a regular basis (remotely between visits and/or at visits), with reminders sent by email, text or automated phone [18]. Daly et al. demonstrated the feasibility of mobile health interventions to assess daily symptoms in patients with intravenous antineoplastic therapy [19]. Basch et al. showed a significant increase in overall survival with electronic PROs (e-PROs) in patients with metastatic cancer, compared to usual care [20]. Simple approaches to symptom measurement are commonly preferred, allowing daily collection of about ten symptoms, readily interpretable for both patients and providers [21]. The COVID-19 pandemic boosted remote follow-up of outpatients. Consequently, some teams developed e-PROs for cancer patients receiving OAAs [22,23,24,25]. Doolin et al. demonstrated that an e-PRO tool monitoring patient concerns about adherence, cost and toxicities was feasible and shortened the delay to symptom assessment [22].

Few research programs have specifically focused on routine PRO assessment in outpatients treated by OAAs. The aim of the present study was therefore to assess symptoms potentially related to adverse events experienced by cancer outpatients treated by OAAs, using PROs with the implementation of the real-life multidisciplinary Oncoral care plan (for ONCological care for outpatients with ORAL anticancer drugs). The secondary objectives were to assess nurse and pharmaceutical interventions (NPIs) related to these declared symptoms, patient adherence and satisfaction with the program.

## 2. Materials & Methods

### 2.1. Patient Cohort

Adult cancer outpatients starting treatment by OAA in the Lyon Sud university hospital of the Hospices Civils de Lyon (France) who were followed in the real-life Oncoral program were eligible. Oncoral is a multidisciplinary program, based on interviews with a pharmacist and a nurse after consultation with the hospital oncologist. It includes a first education session on understanding the prescribed treatment, the goals of oral chemotherapy and how to take it, with a medication plan, the management of major side-effects and the prevention of drug–drug interactions. A comprehensive medication review was performed by the clinical pharmacist at the initiation of OAA, listing all drugs taken by the patient, for medication reconciliation, including OAA and related medications prescribed by the oncologist, and also drugs prescribed by the general practitioner or specialists in case of other diseases and comorbidities. The need of a pharmaceutical intervention to modify drug use was defined according to the individual patient profile, clinical situation and potential severity of the detected interaction. Supplementary educative sessions on side effects are proposed to the patients, depending on the OAA prescribed and the patient’s possible weaknesses. Between two consultations, the patient can contact the nurse for any question concerning symptoms and can contact the hospital pharmacist about treatment, including suspicion of an adverse effect, taking or stopping a drug, and potential interactions.

Adult patients with cancer for which an OAA is prescribed, i.e., cytotoxic agent, targeted therapy, or hormonal therapy (excluding adjuvant treatments), with ambulatory status (not hospitalized) and affiliated to the social security scheme or equivalent could be included in the program. Patients under 18 years of age or adult patients in an institution or under guardianship or protected by the law, without sufficient autonomy for the management of medication at home, and patients treated with adjuvant hormonal therapy or an OAA in a clinical trial or for compassionate use were excluded. Patients, who could not speak and read French or who presented with a cognitive disorder were excluded from the study. Patients were identified at the OAA initiation consultation.

A prospective study on this Oncoral routine program was implemented, to assess the variation of symptoms during follow-up using PROs, from 17 September 2018 to 26 May 2019, with an inclusion period of 24 weeks. Patients were followed up prospectively for 12 weeks for the specific purposes of the study and as needed as part of the Oncoral program.

All patients provided written informed consent to the processing of their personal data, in accordance with the provisions of the French Law n° 78-17 of 6 January 1978 (as modified by Law 2018-493 of 20 June 2018). The study was registered on the National Data Protection Commission register authorized for Hospices Civils de Lyon (n° 15-122).

### 2.2. Symptoms Questionnaire

The symptoms questionnaire used to collect PROs was derived from the validated French version of the NCI PRO-CTCAE item library v1.0 [17]. The PRO-CTCAE was developed to characterize frequency, severity and interference for 78 symptomatic toxicities in adult outpatients undergoing chemotherapy or radiotherapy. Each item was assessed on a 5-point scale for one or more distinct attributes: frequency (f), severity (s), interference with usual or daily activities (i), and/or presence/absence (p). Severity levels (from 0 to 4) were none/mild/moderate/severe/very severe, and frequency levels (from 0 to 4) were never/rarely/occasionally/frequently/almost constantly.

Symptom selection was based on a synthesis between (1) a review of the scientific literature on outcomes commonly used in PROMs designed for cancer patients and (2) real-life feedback on symptoms reported in the Oncoral program. Four studies using 1 to 24 items from the NCI PRO-CTCAE measurement system were identified in the literature on 22 December 2018 using the following terms: “neoplasms/drug therapy” and “patient-reported outcome measures” [26,27,28,29]. Based on these studies and data from the Oncoral program, the study questionnaire rated 15 symptoms: general pain (s); fatigue (s); constipation (s); decreased appetite (s); rash (s); insomnia (s); memory or concentration (s); anxious or discouraged or sad (s); numbness and tingling (s); cough or shortness of breath (s); blurred vision (s); painful urination (s); nausea (f); vomiting (f) and; diarrhea (f). Patients could assess a 16th symptom in an additional free text field at the end of the questionnaire.

### 2.3. Patient Follow-Up

Home self-reporting used a questionnaire included in the patient’s Oncoral diary, which was designed to be completed as quickly and easily as possible. At the initial consultation, the Oncoral team—consisting of a nurse and a pharmacist—trained patients in treatment, symptoms and side-effects management, introduced them to the self-reporting program and helped them to fill out a baseline self-report.

**Figure 1:**
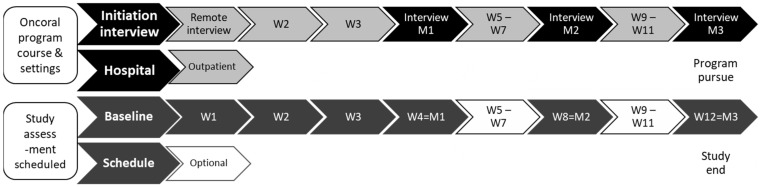
Diagram of patient follow-up in the Oncoral program (all patients) and study assessment schedule (included patients). Caption: W = week; M = month.

Patients were encouraged to self-report symptoms between consultations: once a week during the first month following treatment initiation, and then at the end of the second and third month (baseline; week 1 = W1; week 2 = W2; week 3 = W3; week 4 = W4 or month 1 = M1; week 8 = W8 or month 2 = M2; week 12 = W12 or month 3 = M3) (Figure 1). They could also perform additional voluntary self-assessments at any other time during the study. They were asked to alert a referral nurse or pharmacist, by phone or e-mail, whenever a self-reported symptom reached level 3 or 4 or if they reported >2 level 1 or 2 symptoms. According to PRO-CTCAE, level 0 corresponds to no symptoms and levels ≥3 to severe symptoms. In the present study, PRO levels 1 and 2 corresponded to low and medium symptom levels, respectively, and levels 3 and 4 to high and very high levels. Customized NPIs were performed according to symptom and grade. The self-assessed PRO level could be upgraded or downgraded by the education team, either remotely or during an educational interview in the hospital. NPIs might be health and nutrition advice, symptom management, medication review, care coordination between healthcare professionals, or facilitation of compliance with the customized medication plan.

### 2.4. Outcome Measures

Symptoms type and distribution throughout the study period were assessed at each self-report time-point. NPIs were recorded in a dedicated register throughout follow-up according to a standardized classification of the type of problem encountered and intervention performed.

Adherence to the self-report program was assessed by calculating the study participation rate, defined as the rate of scheduled self-reports completed overall and month by month. The number of unscheduled spontaneous self-assessments was also monitored.

Patient satisfaction with symptom follow-up was assessed in a sample of patients using a satisfaction survey comprising feasibility, reliability and perceived impact on medical care. The purpose was to decide on the continuation of PROs follow-up. The sample comprised a third of the population, i.e., the first 19 patients who had a scheduled medical consultation following the end of the study.

### 2.5. Statistics

Descriptive statistics (i.e., numbers and percentages for categorical variables; mean and range for continuous variables) were used to detail baseline characteristics of the population (age, gender, cancer type, OAA, polypharmacy and comorbidity). Baseline characteristics were extracted from patient medical records and interviews with patients. Polypharmacy is most commonly defined as the use of five or more medications daily [30]. Comorbidity was scored by the referring oncologist on the combined age-Charlson comorbidity index (CA-CCI).

Research questions were: (1) Did the number of symptoms assessed per patient vary during follow-up? (2) For each of the 15 symptoms studied, did the level vary during follow-up? (1) The median number of self-assessed symptoms per patient was compared using a Wilcoxon signed-rank test between baseline and post-baseline evaluations and between first and subsequent months of follow-up in paired series. Post-baseline assessments corresponded to scheduled assessments performed after treatment initiation on M1, M2 and M3. (2) The same comparisons were made with the average level of each of the 15 PROs assessed by all participants. The tests were conducted on smaller samples than the number of patients included in the study because of missing data, which was related to the reporting rate. No strategy for managing missing data was implemented. We considered a two-sided *p*-values < 0.01 for statistical significance to take into account multiple comparisons (Bonferroni correction). Statistical analyses were performed using R software, version 4.1.1 (R Foundation for Statistical Computing, Vienna, Austria).

## 3. Results

### 3.1. Baseline Characteristics

**Figure 2:**
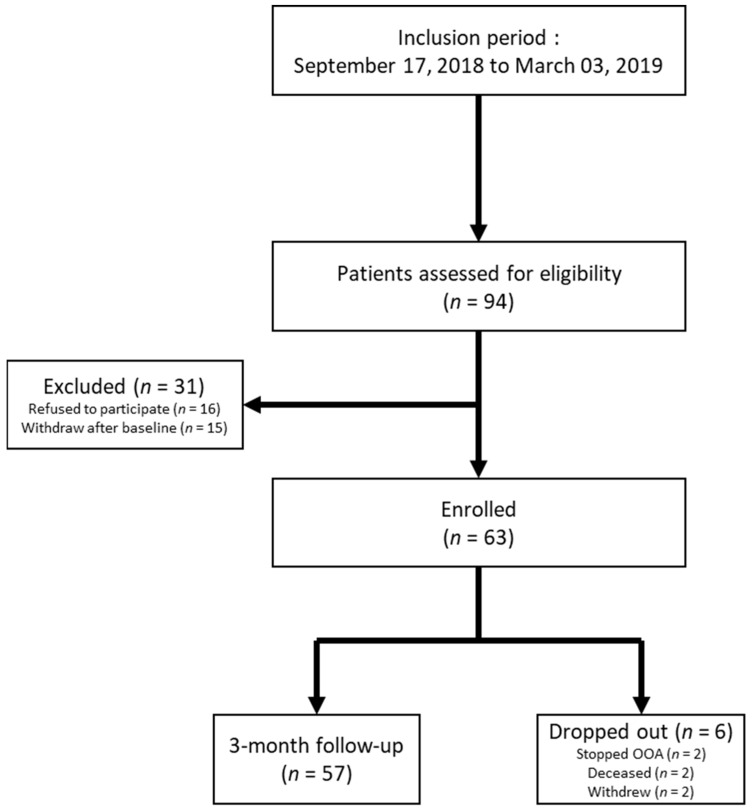
Study Flowchart. Caption: *n* = number of patients.

Between 17 September 2018 and 3 March 2019, 94 patients were eligible for the study. Sixteen declined to participate and 15 withdrew after the baseline assessment. The remaining 63 patients were included; 57 of these completed the study, and 6 were lost to follow-up during the three months of the study (Figure 2).

Baseline characteristics are summarized in Table 1. Mean age was 71 ± 11 years; 49 (77.8%) were over 65 years old, and almost half (47.6%) were over 75 years old. Over half of the participants (57.1%) were female. Median CA-CCI was 3. Polypharmacy concerned 36.5% of patients at OAA initiation. Two-thirds of the patients (*n* = 41) were treated for hematological malignancies, and the others (*n* = 22) for solid cancers. There were 24 different treatment regimens. Most patients were receiving a single OAA (ibrutinib, 20.6%; lenalidomide, 12.7%; palbociclib, 11.1%; niraparib, 6.3%; other, 31.8%) and 11 were receiving a combination of two OAAs (dabrafenib/trametinib, 6.3%; ixazomib/lenalidomide, 4.8%; other, 6.4%).

### 3.2. Reported Symptoms

During the study period, 407 questionnaires were completed and 6,189 PROs were reported, including 2333 symptoms and 3856 outcomes corresponding to no symptoms.

**Figure 3:**
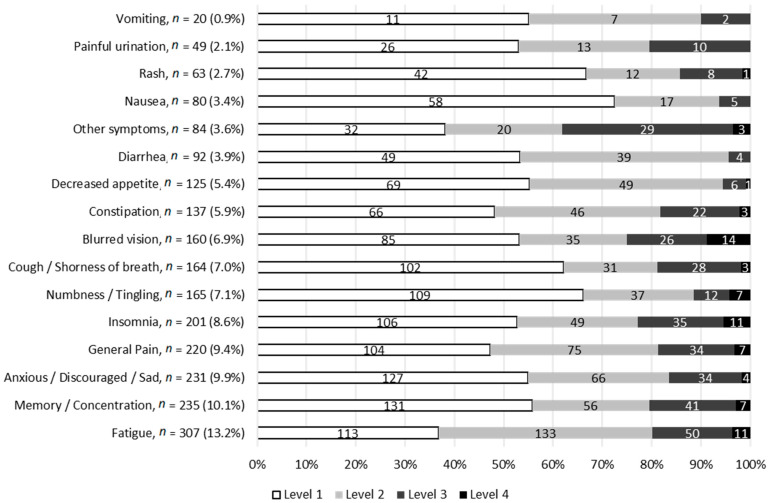
Number of symptoms reported by type and level. Caption: *n* = number of times a symptom was reported during follow-up; level 1 = mild or rarely; level 2 = moderate or occasionally; level 3 = severe or frequently; level 4 = very severe or almost constantly.

The symptoms most commonly experienced were fatigue (13.2%), memory and concentration impairment (10.1%), anxiety, discouragement or sadness (9.9%), general pain (9.4%) and insomnia (8.6%) (Figure 3). Most patients reported one or more episodes of fatigue (92.1%) and general pain (82.5%), as well as psychological disorders; insomnia for eight in ten patients, anxiety, discouragement or sadness for more than three-quarters, and memory and concentration impairment for almost seven in ten (Table 2). Digestive disorders also affected a large majority of patients at least once during the study period: constipation (57.1%), decreased appetite (52.4%), diarrhea (47.6%), nausea (47.6%), or vomiting (14.3%). In addition, the majority of patients reported various other symptoms at least once: cough or shortness of breath (65.1%), numbness or tingling (63.5%), blurred vision (58.7%), rash (42.9%), or painful urination (22.2%) (Table S1). Twenty-three patients reported 34 additional symptoms, 18 of which were already listed on the PRO-CTCAE list; most were specific pains (joint or muscle pain), heartburn, bruising, hot flushes, or cutaneous disorders (dry skin, itch). The other 16 additional symptoms were not on the PRO-CTCAE list and all but one were reported by a single patient each.

Overall, 52.7% of symptoms were categorized as level 1 (mild or rare), 29.4% as level 2 (moderate or occasional), 14.8% as high-level (3, severe or frequent) and 3.1% as very high-level (4, very severe or almost constant) (Figure 3). Almost half of self-reports (42.5%) included at least one high-level symptom. High-level symptoms were experienced by 74.6% of patients during the study period. Visual disorder and blurred vision were the most severe symptoms, assessed as high-level in a quarter of cases, without association with any particular drug.

An average of 5.7 symptoms (range, 0–13) were reported per self-assessment; only one patient reported no symptoms in one questionnaire. There was no significant difference in the median number of self-reported symptoms per patient between baseline and post-baseline assessments (Table S2). After an increase in the number of patients reporting each of the symptoms during the first month of follow-up compared with baseline, the number decreased during the second and third months for all symptom levels taken together and for high and very high levels taken separately (Table 2 and Table S1).

Moreover, there was a trend toward a decrease in the reported level of each of the 15 symptoms between the baseline or M1 and the subsequent months of follow-up; the trend was statistically significant for fatigue between the baseline and M1 (*p* = 0.01) and anxiety, discouragement or sadness between baseline and M1 (*p* = 0.008) (see Table 3). Pairwise comparisons made for the other symptoms were not significant (see Table S3).

### 3.3. Nurse and Pharmacist Interventions (NPIs)

According to the initial instructions provided to patients regarding when to contact the Oncoral team (whenever they experienced ≥1 level 3 or 4 symptoms or ≥3 level 1 or 2 symptoms), 349 self-assessments (85.7%) should have led to contact, but, in fact, only 139 did so, i.e., the compliance rate was 39.8%.

During the study period, there were 317 exchanges between patients and the Oncoral team (telephone call, 56.8%; consultation, 38.8%; e-mail, 4.4%), leading to 264 NPIs (medication review, 31.1%; symptom management, 30.3%; care coordination, 23.5%; therapeutic compliance training, 14.4%, missing data, 0.7%). Fifty-three contacts did not require an NPI.

**Figure 4:**
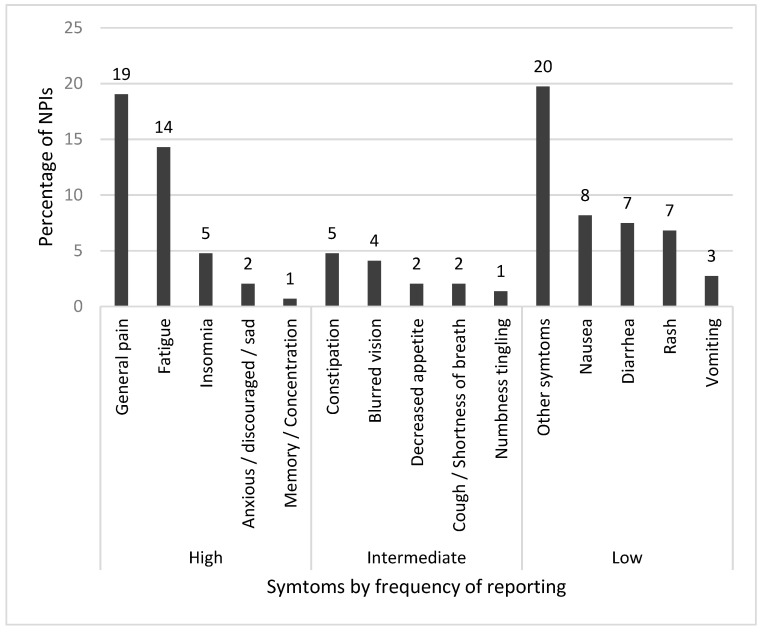
Nurse and pharmacist intervention (NPI) distribution per symptom and per symptom reporting frequency. Caption: High frequency: symptom reported ≥ 200 times during the study period; intermediate frequency: symptom reported 100–199 times during the study period; low frequency: symptom reported < 100 times during the study period.

Sixty-two patients had ≥1 NPIs; the mean number of NPIs per patient was five (range, 0-20). The most common symptoms resulting in an NPI were digestive disorders (constipation, diarrhea, nausea) (20.5%), general pain (19.0%), fatigue (14.3%), rash (6.8%), and insomnia (4.8%). Symptoms rarely reported by patients during the study also led to several NPIs: nausea, diarrhea and rash represented 10.0% of patient-reported symptoms but 22.4% of NPIs. On the contrary, frequently reported symptoms did not systematically lead to an NPI: anxiety, discouragement, sadness and memory or concentration impairment represented 20.0% of patient-reported symptoms, but only 2.7% of NPIs (Figure 4).

### 3.4. Self-Reporting Program Adherence and Patient Satisfaction

The reporting rate was 78.7%; 18 patients shown full adherence to follow-up (100% of scheduled self-assessments completed) during the three-month study period. Over 80% of patients completed at least two-thirds of the scheduled self-assessments. The reporting rate was high in the first four weeks of follow-up: 95% in the first week, 90% in the second week, and 84% in the third and fourth weeks. Rates were lower for the second and third month scheduled self-reports: respectively, 50% and 44%. Twenty-three patients (36.5%) made spontaneous reports in the first and second months and 11 (17.5%) in the second and third months.

**Figure 5:**
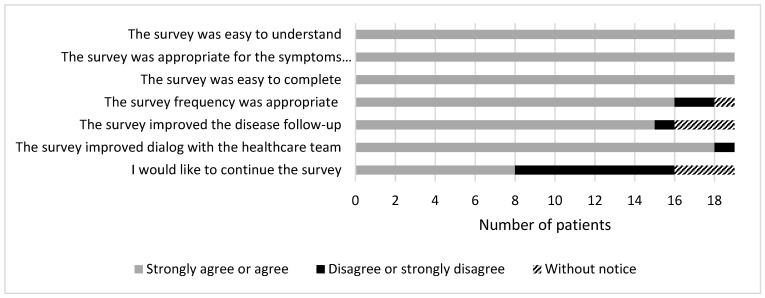
Patient satisfaction survey responses (*n* = 19).

Satisfaction was assessed in a sample of 19 patients. They were very satisfied with follow-up (Figure 5). Regarding content, all patients (100%) felt that the questionnaire was easy to use and appropriate for describing the experience of symptoms. Regarding feasibility, all patients felt it was easy to fill out (100%) and most (84%) considered the follow-up frequency to be appropriate. Regarding the perceived impact on medical care, most patients reported that PRO monitoring improved follow-up (79%) and dialog with healthcare providers (95%). Feedback about willingness to continue the survey was not unanimous, as 42% of patients said they would not continue the survey (*n* = 8); five of these explained that they did not need to continue because they did not experience symptoms during the study period, and therefore had not completed the second and third month self-assessments.

## 4. Discussion

Few previous studies have used PROs to assess the symptoms experienced by outpatients taking OAAs, except for quality-of-life surveys. In the present study, the symptoms reported were of the same type as in previous studies [22,23,24,31]. Most were physical symptoms that are well known to patients and caregivers and easy to recognize and describe, such as cutaneous signs. Other frequently reported symptoms were more subjective psychological symptoms, such as stress, anxiety and sadness, which are less noticeable to caregivers and more difficult for patients to admit. PROs facilitate reporting psychological disorders, and thus facilitate their management by health professionals. One of the strengths of PROs is that they reveal symptoms that are potentially undetected in or between consultations.

In some studies, the symptoms reported by PROs were mostly moderate, suggesting under-reporting of serious symptoms that would require urgent management. Thus Mackler et al. found almost exclusively mild-to-moderate symptoms (94.4%) in patients treated by OAAs, using the revised Edmonton Symptom Assessment Scale (ESAS-r) [31]. Basch et al. found 1.7% severe or disabling symptoms in outpatients receiving chemotherapy for advanced solid tumor [32]. In the present Oncoral study, although most PROs were mild or moderate (level 1 or 2), 17.9% were high or very high level (level 3 or 4). Moreover, at least one high-level symptom was reported in half of the questionnaires and by three-quarters of patients. The symptoms reported at baseline were probably due to cancer and/or previous lines of treatment, while symptoms reported during the first month could also be due to the OAA. The decrease in the number and severity or frequency of symptoms reported in months two and three could be due to spontaneous regression, medication overlap at initiation, the benefit of the Oncoral program follow-up, or patients’ weariness with self-reporting. Early PROs reporting potentially serious symptoms were consistent with previous reports. Nachar et al. observed a similar frequency, with 46% of assessments including at least one severe symptom in a comparatively elderly cohort [24].

In the present study, the level of symptoms leading to most NPIs (general pain, fatigue, digestive disorder, insomnia) decreased over follow-up; the difference was significant for insomnia and fatigue. A trend test is generally more suitable than pairwise comparisons. However, few patients systematically self-assessed their symptoms at baseline and at each month of follow-up. Additionally, a trend test might not only have been biased because it was limited to a specific subgroup, but also underpowered. The multiplication of pairwise comparisons exposed the risk of a statistically significant test by chance. However, the Bonferroni correction is known to be conservative, and applying a per-result correction to correct for significant false *p*-values was considered an acceptable option. Therefore, the results should be interpreted with caution. The study has other limitations. Patient characteristics were not been studied as potential confounders. In addition, other variables that may influence individuals’ responses to the PROs questionnaires, i.e., race, education, and income, were not studied. Nevertheless, the results encourage conducting a randomized study to assess prolongation of overall survival with PROs/ePROs, as observed in certain cancers. Further research is also needed to investigate the association between the Oncoral program, NPIs and PROs. Three studies evaluating the clinical and medico-economic impact of the Oncoral program are underway (NCT03660670, NCT03257969, NCT02849535). Patients who experienced a great number of high-level symptoms received a greater number of NPIs. Although further studies are needed for confirmation, these results encourage combining of educational follow-up, such as the Oncoral program with early symptom reporting using PROMs. The strength of the present study and its originality is that PROs were part of an outpatient education program. The education sessions carried out by the nurse and pharmacist in the framework of the real-life Oncoral program allowed patients to acquire the knowledge and skills needed to identify symptoms, including the most serious ones, which should lead them to contact their oncologist [33]. This education and training enhances PRO reporting, in terms of both frequency and severity. In the future, the use of PROMs should be integrated into educational programs to reinforce patient safety.

In addition, although symptom severity varies in the literature, these results appear to confirm the trend of self-reported symptoms being very numerous [34]. Patients receiving OAAs had a substantial symptom burden, showing that quality of care could be improved by focusing providers’ attention on the symptoms that affect patients’ quality of life [31,35]. Several studies showed that routine collection of PROs enhances patient empowerment, patient and provider communication, patient satisfaction, treatment monitoring, detection of unrecognized problems, symptom management and treatment adherence [32,36,37,38,39,40,41,42,43]. Not all included patients were asked about their satisfaction because the responses of the first patients were homogeneous and favorable to the continuation of PRO follow-up. Since the start of the study in 2018, PRO follow-up has remained an integral part of the Oncoral program. Moreover, PRO assessment may be prognostic for medical consultation, hospital admission and cancer survival [44,45]. The Setting International Standards in Analyzing Patient Reported Outcomes and Quality of Life Endpoints Data Consortium has recently developed a set of recommendations to facilitate standard approaches for PRO analysis [46]. Beyond PRO measurement and satisfaction surveys, patient-reported experience measures (PREMs) should be widely used in the future to evaluate healthcare services, increase the central role of patients in healthcare decision-making and improve quality of care [47].

The most frequently reported symptoms in this study were fatigue, general pain and psychological disorders, such as insomnia, anxiety, discouragement, sadness or impaired memory or concentration. In contrast, some symptoms that might be expected with injectable chemotherapy were usually mild or absent. OAAs frequently concerned targeted therapies, which are generally better tolerated and more comfortable than injectable cytotoxic drugs [48]. In choosing both physical and psychological criteria, we showed that patients taking OAAs had a high symptom burden. Psychological distress, which has been associated with higher rates of cancer mortality, is an important area and should be more thoroughly documented [49]. Several symptoms, such as anxiety, sadness, discouragement, or memory and concentration impairment, were deemed relevant by patients but seemed to be underestimated; although accounting for 20.0% of patient-reported symptoms, only 2.7% of them led to an NPI. Concordance of symptom reporting rates between patients and physicians may be greater for outwardly observable symptoms than for subjective symptoms [6,7,50,51]. The present study encourages associating an onco-psychologist to the pharmacist and nurse in the Oncoral follow-up. Frequent and/or severe psychological disorders reported on PROMs should systematically lead to the patient being referred to a supportive care consultation.

In the present study, only 23% of self-assessments that should have led to contact actually resulted in an NPI related to symptom management. Some hypotheses may explain this. Firstly, symptoms may have been downgraded by the Oncoral team; a specific guidance document appears to be essential, to harmonize responses to patients’ alerts. Secondly, intervention may have been conducted by other health professionals, such as the general practitioner or the community pharmacist or more likely by the OAA physician or medical oncology team, especially for severe symptoms. Reinforcing the involvement of all healthcare professionals in the Oncoral program should improve symptom reporting and care coordination. Thirdly, some patients may have hesitated to contact the Oncoral team. One limitation of the present study was that the rate of compliance with the contact process was low, at 39.8%. In the light of the available data, it seems possible to further optimize the procedure. On the one hand, paper-based PRO assessment is limited by a delay in generating clinically meaningful interpretations [52]. A digital interface between patient and healthcare professionals, with a real-time alert system, could overcome these difficulties. On the other hand, the conditions triggering contact with the education team are to be re-evaluated. In the present study, because of the high reporting rate, contact instructions provided to the patient should be more specific, to be more effective, only addressing patients to the hospital team in the case of high-level symptoms. Mild and moderate symptoms could be managed directly via a digital interface with a validated guidance algorithm, or by community health professionals closer to the patients, provided they are trained, involved and motivated. In addition, it is likely that, in the present study, the paper diary may have lacked reactivity and user-friendliness, discouraging self-reporting after the first month of follow-up. Digital tools would undoubtedly increase the reporting rate. The integration of a digital tool for remote monitoring and patient-reporting has become essential to secure oral chemotherapy in outpatients, enhance dialogue between patients and healthcare professionals and provide a link between hospital and community nurses, pharmacists and physicians.

More and more local initiatives are trying out digital tools to manage patients treated by OAAs, to improve drug toxicity detection and overall survival. Recently, Collado-Borrell et al. tested several health outcome improvements for drug-related problems, side-effects, adherence, etc., using a specific smartphone app [23]. During the COVID-19 pandemic, Doolin et al. demonstrated the efficacy of e-PRO monitoring in a population of 62 cancer outpatients treated by OAAs, with a delay in reporting first symptoms of three days in the e-PRO group, versus seven days in the historical comparison group, leading to treatment delays of eight and fourteen days respectively [22]. Although e-PROs have proved to be effective in securing cancer patients [22,23,32], it is still unclear whether they provide sufficient benefit in relation to cost and the burden on health professionals due to supplementary workload [53]. For example, Doolin et al. showed no significant difference between an e-PRO monitoring group and a historical comparison group for 30-day or 90-day emergency department consultation or admission (respectively, 12% for both groups, and historical 28% and e-PRO 20%) [22]. It should be highlighted that improved survival has been demonstrated with the use of e-PROs in recent studies [20,32,54,55]. We recommend incorporating e-PROs in outpatient educational follow-up programs, such as Oncoral. This helps educate the patient in recognizing reportable symptoms and promptly identifying the serious signs that should lead to immediate contact with the oncologist. It is essential to combine the two approaches to improve early reporting of symptoms and early management by health professionals, guaranteeing the safety of cancer outpatients taking OAAs.

One of the major challenges of integrating patient self-reporting systems into clinical practice is to actively involve community and hospital healthcare professionals, which requires close coordination. Even if most of them agree that hearing the patient’s voice is necessary, it is less clear how best to integrate patient self-reporting into the existing clinical care workflow without overburdening the professionals [56]. A formal economic evaluation should be performed to provide evidence about the benefits of PRO assessment in oncologic clinical practice. Last but not least, the management of patients on OAAs must progress towards more personalized follow-up, with a particular emphasis on symptoms that are often neglected (fatigue, concentration problems, etc.) but which are no less important to monitor in order to improve patients’ quality of life.

## 5. Conclusions

In view of the trend toward patient-centered care, the use of PROs appears essential to improve clinical decision-making. This initiative is the first step in the implementation of symptom assessment by PROs in patients treated by OAAs. To incorporate this program in a digital interface may be useful for patients and providers, and could encourage symptom reporting, enhance communication between patients and healthcare professionals, and improve identification of symptom burden and quality of care. The stakes are high. With early self-reporting of symptoms by patients, proactive management by health professionals is expected, which should lead to improved survival.

## Figures and Tables

**Table 1:** Patient Baseline Characteristics.

Characteristics	Mean (Range)	Number of Patients	% of Patients
**Age (** **years** **)**	71 (41–89)		
≥65 years		49	77.8
≥75 years		30	47.6
**Female gender**		36	57.1
**Cancer type**			
*Hematological malignancy*		41	65.1
Multiple Myeloma		14	22.2
Chronic Lymphoid Leukemia		10	15.9
Lymphoma		5	7.9
Other		12	19.1
*Solid cancer*		*22*	*34.9*
Breast cancer		7	11.1
Ovarian cancer		6	9.5
Melanoma		4	6.4
Other		5	7.9
**Oral Anticancer Agent**			
*One-agent regimen*		52	82.5
Ibrutinib		13	20.6
Lenalidomide		8	12.7
Palbociclib		7	11.1
Niraparib		4	6.3
Other		20	31.8
*Two-* *agentregimen*		11	17.5
Dabrafenib + Trametinib		4	6.3
Ixazomib + Lenalidomide		3	4.8
Other		4	6.4
**Polypharmacy at Oral Anticancer Agent initiation**			
≥5 daily medications,		23	36.5
Mean number of daily medications (range)	5 (1–11)		
**Combined Age Charlson Comorbidity Index (CA-CCI) score at Oral Anticancer Agent initiation**	3 (0–7)		
≤1 point		9	14.3
2 points		10	15.9
3 points		18	28.6
4 points		13	20.6
≥5 points		11	17.4
Missing data		2	3.2

**Table 2:** Symptoms reported by more than half of patients at baseline: number of patients reporting these symptoms overall, at baseline and during each month of follow-up.

	Number of Patients (*n* = 63)									
	Overall		Baseline		1st Month		2nd Month		3rd Month	
Levels	All	3 & 4	All	3 & 4	All	3 & 4	All	3 & 4	All	3 & 4
Fatigue	58	28	51	15	56	20	30	7	22	4
General Pain	52	17	40	10	47	13	19	4	12	2
Insomnia	50	14	34	8	42	12	18	5	13	1
Anxious/Discouraged/Sad	48	14	42	10	41	8	24	7	19	4
Memory/Concentration	42	13	39	9	38	11	23	4	17	6

Levels 3 & 4 = severe to very severe symptom or frequently to almost constantly experienced symptom.

**Table 3:** Symptoms with a reported median level greater than or equal to 1: median (range) level of each symptom and comparison between baseline and M1 of follow-up.

Symptoms	Baseline	1st Month	*p* Value (*n* = 52)
Fatigue	2.0 (0–4)	1.0 (0–4)	0.01
Memory/Concentration	1.0 (0–4)	1.0 (0–4)	0.532
Anxious/Discouraged/Sad	1.0 (0–4)	1.0 (0–3)	0.008
General Pain	1.0 (0–4)	1.0 (0–3)	0.179
Insomnia	1.0 (0–4)	0.5 (0–4)	0.308

*n* = number of patients.

## Data Availability

The data that support the findings of this study are available from the corresponding author, upon reasonable request.

## Supplementary Materials

The following supporting information can be downloaded at: https://www.mdpi.com/article/10.3390/cancers14030660/s1, Table S1: Number of patients reporting each symptom overall, at baseline and during each month of follow-up, Table S2. Median (range) number of symptoms assessed per patient and comparison between baseline or M1 and subsequent months of follow-up, Table S3. Median (range) level of each symptom and comparison between baseline or M1 and subsequent months of follow-up.
