# Supplementary material for: Assessment of Patient Reported Outcomes (PROs) in Outpatients Taking Oral Anticancer Drugs Included in the Real-Life Oncoral Program
Source: Cancers (Basel). 2022 Jan 28;14(3):660. doi: 10.3390/cancers14030660 (PMC8833408; doi:10.3390/cancers14030660)
Supplement: Supplementary file 1 [file cancers-14-00660-s001.zip › cancers-1570887-supplementary.pdf]

cancers

MDPI

Table S1. Number of patients reporting each symptom overall, at baseline and during each month of follow-up.

	Number of patients (n = 63)									
	Overall		Baseline		1st month		2nd month		3rd month	
Levels	*All*	*3 & 4*	*All*	*3 & 4*	*All*	*3 & 4*	*All*	*3 & 4*	*All*	*3 & 4*
Fatigue	58	28	51	15	56	20	30	7	22	4
General Pain	52	17	40	10	47	13	19	4	12	2
Insomnia	50	14	34	8	42	12	18	5	13	1
Anxious / Discouraged / Sad	48	14	42	10	41	8	24	7	19	4
Memory / Concentration	42	13	39	9	38	11	23	4	17	6
Cough / Shortness of breath	41	12	25	3	34	10	20	7	11	2
Numbness / Tingling	40	6	25	3	32	5	21	1	11	1
Blurred vision	37	9	18	5	32	9	18	5	13	5
Constipation	36	12	25	6	33	10	16	2	9	3
Decreased appetite	33	5	20	2	26	5	13	0	9	0
Diarrhea	30	4	15	1	24	2	6	1	5	0
Nausea	30	5	10	1	27	4	9	0	5	0
Rash	27	5	7	0	19	5	8	1	6	1
Painful urination	14	2	7	1	11	2	8	2	4	1
Vomiting	9	2	4	0	7	2	3	0	1	0
Other	23	9	7	3	21	9	11	4	7	2

Levels 3 & 4 = severe very severe symptom or frequently to almost constantly experienced symptom.

Table S2. Median (range) number of symptoms assessed per patient and comparison between baseline or M1 and subsequent months of follow-up.

	B	M1	*p* value (n=52)	B	M2	*p* value (n=29)	B	M3	*p* value (n=25)	M1	M2	*p* value (n=29)	M1	M3	*p* value (n=25)
All levels	5.5 (0-11)	5.0 (1-11)	0.392	5.0 (0-10)	6.0 (0-10)	0.332	5.0 (0-10)	6.0 (0-9)	0.487	5.0 (1-11)	6.0 (0-10)	0.288	5.0 (1-11)	6.0 (0-9)	0.311
Levels 1-2	5.0 (0-10)	5.0 (0-11)	0.951	5.0 (0-8)	4.0 (0-10)	0.736	5.0 (0-8)	4.0 (0-9)	0.610	5.0 (0-11)	4.0 (0-10)	0.495	5.0 (0-11)	4.0 (0-9)	0.418
Levels 3-4	1.0 (0-6)	0.0 (0-5)	0.095	0.0 (0-6)	0.0 (0-6)	0.302	0.0 (0-6)	0.0 (0-7)	0.858	0.0 (0-5)	0.0 (0-6)	0.927	0.0 (0-5)	0.0 (0-7)	0.930

B = Baseline; M1 = Month 1; M2 = Month 2; M3 = Month 3.

Table S3. Median (range) level of each symptom and comparison between baseline or M1 and subsequent months of follow-up.

	B	M1	*p* value (n=52)	B	M2	*p* value (n=29)	B	M3	*p* value (n=25)	M1	M2	*p* value (n=29)	M1	M3	*p* value (n=25)
Fatigue	2.0 (0-4)	1.0 (0-4)	0.010	1.0 (0-4)	1.0 (0-4)	0.098	1.0 (0-4)	1.0 (0-4)	0.968	1.0 (0-4)	1.0 (0-4)	0.276	1.0 (0-4)	1.0 (0-4)	0.331
Memory / Concentration	1.0 (0-4)	1.0 (0-4)	0.532	1.0 (0-3)	1.0 (0-3)	1.000	1.0 (0-3)	1.0 (0-3)	1.000	1.0 (0-3)	1.0 (0-3)	0.299	1.0 (0-3)	1.0 (0-3)	0.188
Anxious / Discouraged / Sad	1.0 (0-4)	1.0 (0-3)	0.008	1.0 (0-4)	1.0 (0-3)	0.188	1.0 (0-4)	1.0 (0-3)	0.080	1.0 (0-3)	1.0 (0-3)	0.759	1.0 (0-3)	1.0 (0-3)	0.824
General Pain	1.0 (0-4)	1.0 (0-3)	0.179	1.0 (0-4)	0.0 (0-3)	0.306	0.0 (0-4)	0.0 (0-4)	0.193	0.0 (0-3)	0.0 (0-3)	0.521	0.0 (0-3)	0.0 (0-4)	0.330
Insomnia	1.0 (0-4)	0.5 (0-4)	0.308	1.0 (0-3)	0.0 (0-3)	0.158	0.0 (0-4)	0.0 (0-4)	0.549	1.0 (0-4)	0.0 (0-3)	0.095	0.0 (0-4)	0.0 (0-4)	0.565
Numbness / Tingling	0.0 (0-4)	0.0 (0-4)	0.464	0.0 (0-3)	0.0 (0-4)	0.437	0.0 (0-4)	0.0 (0-3)	0.627	0.0 (0-4)	0.0 (0-4)	0.821	0.0 (0-3)	0.0 (0-3)	0.572
Cough / Shortness of breath	0.0 (0-3)	0.0 (0-4)	0.237	0.0 (0-3)	0.0 (0-3)	0.539	0.0 (0-3)	0.0 (0-3)	0.890	0.0 (0-4)	0.0 (0-3)	0.886	0.0 (0-3)	0.0 (0-3)	0.244
Blurred vision	0.0 (0-4)	0.0 (0-4)	0.141	0.0 (0-4)	0.0 (0-4)	0.177	0.0 (0-4)	0.0 (0-4)	0.115	0.0 (0-4)	0.0 (0-4)	1.000	1.0 (0-4)	0.0 (0-4)	0.824
Constipation	0.0 (0-4)	0.0 (0-3)	0.332	0.0 (0-4)	0.0 (0-3)	0.045	0.0 (0-4)	0.0 (0-3)	0.223	0.0 (0-3)	0.0 (0-3)	1.000	0.0 (0-3)	0.0 (0-3)	0.374
Decreased appetite	0.0 (0-4)	0.0 (0-3)	0.740	0.0 (0-4)	0.0 (0-2)	0.813	0.0 (0-4)	0.0 (0-2)	0.608	0.0 (0-2)	0.0 (0-2)	0.773	0.0 (0-2)	0.0 (0-2)	0.665
Diarrhea	0.0 (0-2)	0.0 (0-3)	0.675	0.0 (0-2)	0.0 (0-1)	0.299	0.0 (0-1)	0.0 (0-2)	0.484	0.0 (0-2)	0.0 (0-1)	0.124	0.0 (0-3)	0.0 (0-2)	0.203
Nausea	0.0 (0-3)	0.0 (0-3)	0.259	0.0 (0-2)	0.0 (0-2)	0.520	0.0 (0-1)	0.0 (0-2)	1.000	0.0 (0-2)	0.0 (0-2)	0.341	0.0 (0-2)	0.0 (0-2)	0.048
Rash	0.0 (0-2)	0.0 (0-3)	0.718	0.0 (0-2)	0.0 (0-3)	0.746	0.0 (0-2)	0.0 (0-3)	1.000	0.0 (0-3)	0.0 (0-3)	1.000	0.0 (0-3)	0.0 (0-3)	0.586
Painful urination	0.0 (0-3)	0.0 (0-3)	0.850	0.0 (0-3)	0.0 (0-3)	0.850	0.0 (0-2)	0.0 (0-3)	1.000	0.0 (0-3)	0.0 (0-3)	0.346	0.0 (0-3)	0.0 (0-3)	1.000
Vomiting	0.0 (0-2)	0.0 (0-2)	0.773	0.0 (0-2)	0.0 (0-1)	1.000	0.0 (0-2)	0.0 (0-2)	NA	0.0 (0-2)	0.0 (0-1)	0.346	0.0 (0-2)	0.0 (0-2)	1.000

B = Baseline; M1 = Month 1; M2 = Month 2; M3 = Month 3; NA = not applicable.
